# The impact of eye dominance on fixation stability in school-aged children

**DOI:** 10.16910/jemr.16.3.6

**Published:** 2023-12-31

**Authors:** Evita Serpa, Madara Alecka, Ilze Ceple, Gunta Krumina, Aiga Svede, Evita Kassaliete, Viktorija Goliskina, Liva Volberga, Asnate Berzina, Rita Mikelsone, Elizabete Ozola, Daniela Toloka, Tomass Ruza, Anete Klavinska, Sofija Vasiljeva, Marija Koleda

**Affiliations:** University of Latvia, Riga, Latvia

**Keywords:** eye dominance, fixation stability, bivariate contour ellipse area, eye tracking, school-age children

## Abstract

The aim of the study was to analyze the stability of dominant and non-dominant eye fixations, as well as the influence of development on fixation stability. The study analyzed fixation stability in 280 school-age children, ranging in age from 7 to 12 years old. Fixation stability was determined by calculating the bivariate contour ellipse area (BCEA). During the fixation task, eye movements were recorded using the Tobii Pro Fusion eye tracking device at a 250 Hz sampling frequency. The results indicate that the fixation stability of dominant and non-dominant eyes, as well as the fixation stability of each eye regardless of dominance, improves as children grow older. It was found that for 7 and 8- year-old children, fixation in the dominant eye is significantly more stable than in the non-dominant eye, while in older children, there is no significant difference in fixation stability between the dominant and non-dominant eye.

## Introduction

Fixation stability is the ability to maintain a stable gaze position on a fixation target. Typically, binocular fixation is more stable than monocular fixation (12; 16). In various pathological cases, it is observed that fixation in one eye is more stable. For example, in cases of amblyopia, fixation is less stable in the amblyopic eye (1; 8), whereas in cases of maculopathy, fixation is more stable in the healthier eye (17). Studies comparing fixation stability in individuals without pathologies have shown that fixation stability in the right and left eyes does not significantly differ (8; 19).

It is important to consider that each individual has a dominant eye, which processes sensory information faster and more strongly (18). However, unstable eye dominance can also be observed when the visual system, in binocular viewing, switches preference for which eye's information it prioritizes (4). There have been limited studies on fixation stability in the dominant and non-dominant eye, but they suggest that eye dominance does not play a significant role in fixation stability (16) or that fixation in the dominant eye may be slightly more stable (22). These studies were conducted on adults, and as far as is known, the role of eye dominance in fixation stability has not been studied in the children population.

Fixation stability can be quantitatively assessed using various methods. Thaler et al. (20) evaluated fixation stability by analyzing dispersion and microsaccade rate. The Center of Gravity method is also used, where the average distance of fixations from the fixation center is calculated (3). However, the most common method used in most studies is the calculation of the bivariate contour ellipse area (BCEA) (2; 12; 19). Methodology for analyzing fixation stability may vary in other aspects as well. Some studies focus on fixation stability by selecting the dominant eye (11), while others analyze it by selecting one eye without considering which one is dominant (6; 20). Additionally, there are studies that analyze binocular fixation stability (12).

Research on fixation development suggests that binocular fixation becomes more stable with age (3), and it also changes over the lifespan (2). It is expected that a similar finding may be observed with monocular fixation stability. To determine this, one of the aim of this study is to analyze whether monocular fixation stability improves with increasing age in school-age children.

Since it is not known whether the dominant and non-dominant eyes have a direct impact on fixation stability in children and whether eye dominance should be considered in studies analyzing fixation stability choosing one eye, the main objective of this work is to determine whether there is a difference in fixation stability between the dominant and non-dominant eye in school-age children.

## Methods

### Participants

In the study, a total of 379 school-age children (184 boys and 195 girls) aged between 6 and 13 years old were enrolled. Subsequently, the study included those children whose uncorrected visual acuity monocularly was at least 0.4 (decimal units), meaning that each eye separately should be able to see the optotypes corresponding to a visual acuity of 0.4 on the visual acuity chart. This visual acuity threshold was selected to ensure that the child would be able to see the stimuli on the screen monitor without wearing glasses, if glasses are worn daily. Another inclusion criterion was binocular single vision (assessed with the TNO test).

Using the mirror test, the motor-leading eye in near (40 cm) was determined for the study participants. During the test, participants were asked to look into a mirror and align the fixation target on the mirror with the tip of their nose in the mirror image. The eye for which the fixation target was visible at the tip of the nose, while the other eye was covered, was identified as the motor-dominant eye.

A total of 20 children did not meet the criteria for visual acuity and binocular vision. 79 children did not have a valid eye movement record due to insufficient participation in the measurement or due to technical issues during the measurement, e.g. the data of one eye was not recorded. The total number of children who met the initial selection criteria and had a valid eye movement record for data analysis was 280. The children were then divided into 6 age groups, based on their year of birth and their full age in years at the time of measurement (see Table 1).

**Table 1.** The total number of children and the distribution of dominant eyes in each age group.

Age (years)	Number of children (n)	Dominant eye	
		right	left
7	44	31	13
8	49	34	15
9	47	33	14
10	54	37	17
11	55	41	14
12	31	19	12

The study was approved by the Ethics Committee of the University of Latvia and was conducted in accordance with the Declaration of Helsinki. Children's participation in the study was voluntary, and only those whose parents had provided written permission for their child's involvement took part in the study.

### Procedure

To assess fixation stability, a fixation task was conducted in which a fixation target was presented on a computer monitor located 65 cm away from the study participant. The monitor used had dimensions of 52.70 cm x 29.64 cm and a resolution of 1920 x 1080 pixels. The size of the fixation stimulus was 0.6 degrees, and it was presented on a gray background (RGB: 180, 180, 180). The chosen fixation target consisted of a black circle and a white cross combination. This fixation target was selected based on prior research by Thaler et al. (20), which recognized it as providing the most stable fixation. In our experiment, the fixation target was presented for 10 seconds. In the fixation analysis, the middle 9 seconds were included, excluding the first and last 0.5 seconds of the fixation target demonstration period. The task was performed by fixating on the target in a binocular viewing condition, and during fixation, both eyes were recorded simultaneously.

During the fixation task, eye movements were recorded using the Tobii Pro Fusion eye tracker and the Titta Master toolbox (13). Eye movements were recorded at a sampling frequency of 250 Hz. Before recording the eye movements of each participant, a 5-point calibration procedure was performed monocularly, meaning that each eye was calibrated separately.

### Data analysis

For fixation analysis, the I2MC algorithm was used. This algorithm is designed for processing data characterized by high levels of noise and missing data, making it especially advantageous for children's fixation data (9). The algorithm was used to obtain the value of fixation stability for each eye, expressed as the BCEA (bivariate contour ellipse area) value, which is calculated using the following Equation (Eq. 1):

**Eq.1:** Calculation of bivariate contour ellipse area. σH is the standard deviation of fixations in the horizontal meridian, σV is the standard deviation of fixations in the vertical meridian, and ρ is the Pearson product-moment correlation coefficient between two position components.

$$BCEA=2k\pi \sigma_{H}\sigma_{V}{\left(1-\rho^{2}\right)}^{\frac{1}{2}}$$

In Equation 1 *k* is 1.14, as the chosen probability area is 68% (6). A smaller BCEA value indicates a more stable fixation.

### Statistical Analysis

The statistical data processing and analysis were performed using the SPSS 29.0 software (SPSS Inc., Chicago, IL, USA). To determine whether there is a correlation between fixation stability and age, Spearman's Rank-Order Correlation test was applied. The Shapiro-Wilk normality test was used to assess whether the sample data follow to a normal distribution. To compare fixation stability between each eye within each group, the choice of the test was based on the results of the Shapiro-Wilk normality test. If the data did not conform to a normal distribution, the Wilcoxon signed-rank test was used.

## Results

### Fixation Stability in the Dominant and Non-dominant Eyes in Each Age Group

**Figure 1.**
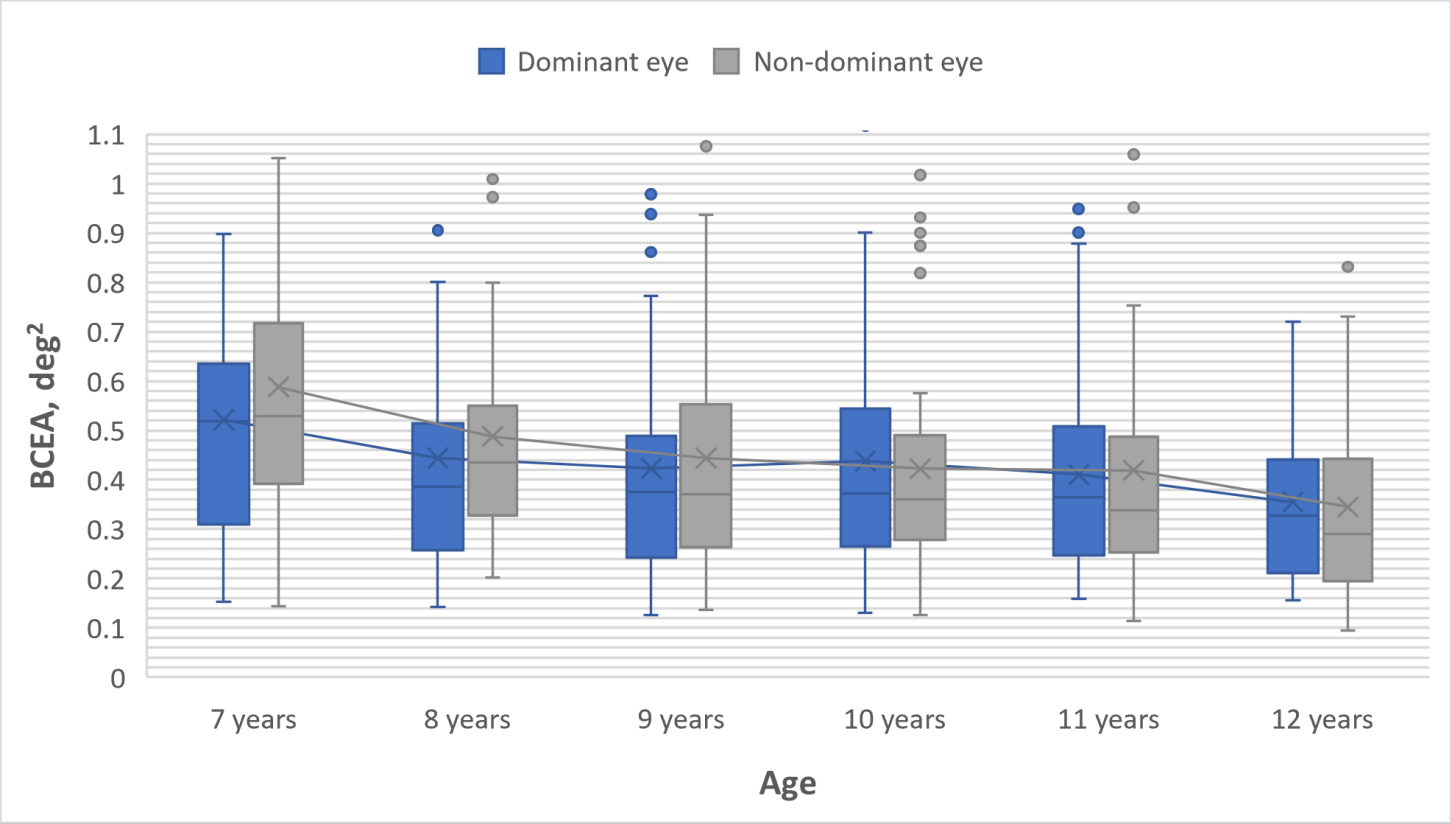
Comparison of gaze fixation stability between the dominant and non-dominant eyes across children's age groups. Box plots present interquartile range, upper on lower whisker, median value marked with a line and mean value marked with a cross. Box plots are connected with trend line.

The Spearman rank correlation coefficient showed that there was a correlation between the children age and fixation stability in the dominant eye (r_S_ = -.181, n = 280, p = .02) and in the non-dominant eye (r_S_ = -.272, n = 280, p < .001). Fixation became more stable with increasing age in both the dominant and non-dominant eye (see Figure 1).

As the Shapiro-Wilk normality test indicated that the measurement values of fixation stability in the study participants did not follow a normal distribution (p < .05), the Wilcoxon signed-rank test was used to compare if there is a significant difference in fixation stability between the dominant and non-dominant eye within each age group. The results demonstrated that fixation in the dominant eye is significantly more stable than in the non-dominant eye for 7-year-olds (Z = -2.101, p = .036) and 8-year-olds (Z = -2.601, p = .009) (see Table 2).

**Table 2.** Fixation stability (mean ± standard deviation) in the dominant and non-dominant eye in each age group. Highlighted are the age groups where a significant difference in fixation stability between the dominant and non-dominant eye was observed.

Age (years)	Dominant eye, BCEA (degrees^2^) ± SD	Non-dominant eye, BCEA (degrees^2^) ± SD	p
7	***0,52 ± 0,26***	***0,59 ± 0,32***	***0,036***
8	***0,44 ± 0,27***	***0,49 ± 0,29***	***0,009***
9	0,42 ± 0,23	0,44 ± 0,25	0,634
10	0,44 ± 0,24	0,42 ± 0,22	0,651
11	0,41 ± 0,24	0,42 ± 0,26	0,880
12	0,40 ± 0,29	0,40 ± 0,34	0,601

### Fixation Stability in the Right and Left Eyes in Each Age Group

**Figure 2.**
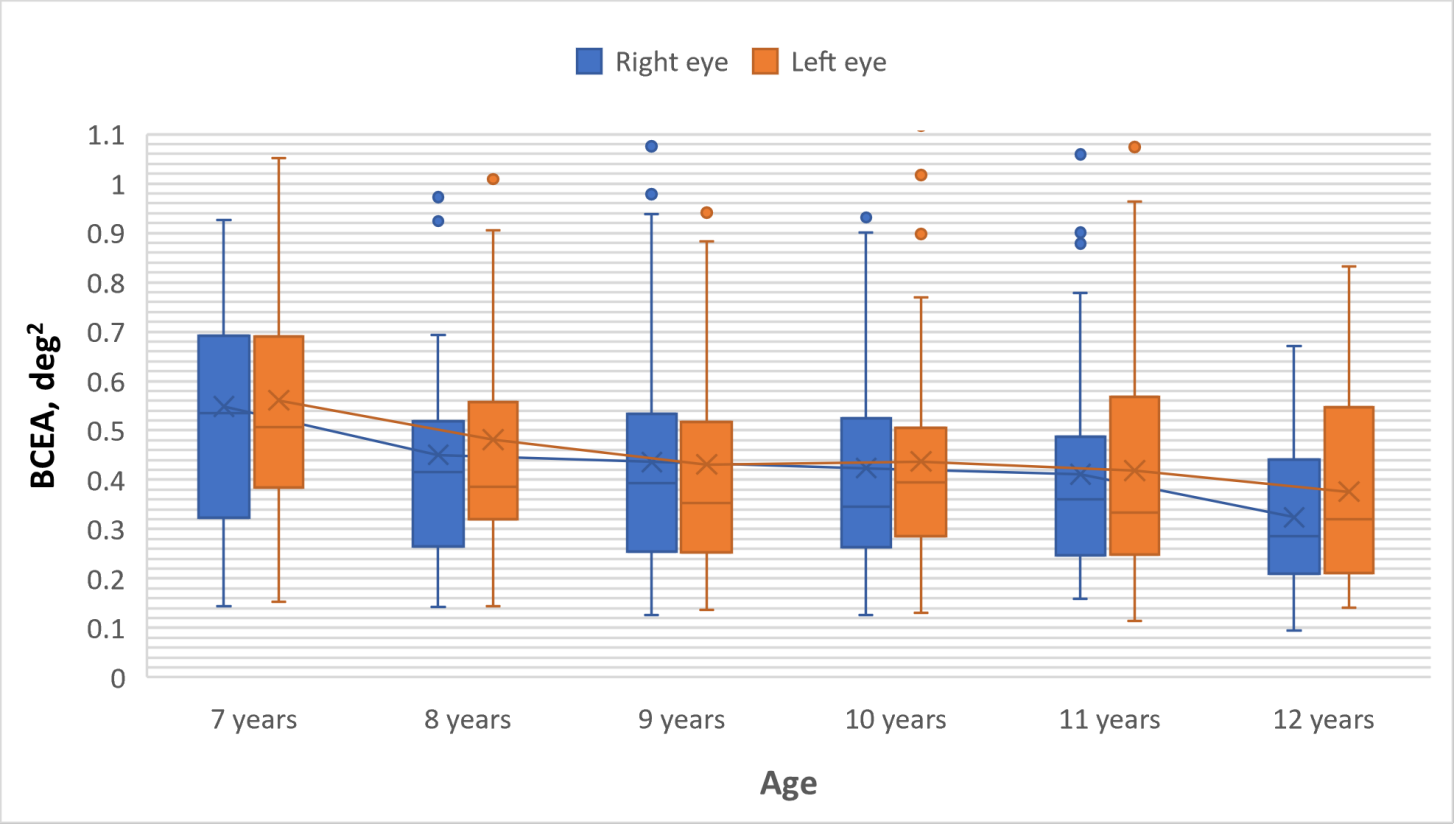
Comparison of gaze fixation stability between the right and left eyes across children's age groups. Box plots present interquartile range, upper on lower whisker, median value marked with a line and mean value marked with a cross. Box plots are connected with trend line.

Comparison was also made between fixation stability in the right and left eyes, without considering eye dominance. Even without considering eye dominance, it was found that fixation stability in both the right eye (r_S_ = -.245, n = 280, p < .001) and the left eye (r_S_ = -.213, n = 280, p < .001) is associated with the child's age, i.e., fixation in both eyes becomes more stable as the child grows older (see Figure 2).

Using the Wilcoxon signed-rank test, it was determined that there is no statistically significant difference in fixation stability between the right and left eyes in any of the age groups (see Table 3).

**Table 3.** Fixation stability (mean ± standard deviation) in the right and left eyes in each age group.

Age (years)	Right eye, BCEA (degrees^2^) ± SD	Left eye, BCEA (degrees^2^) ± SD	p
7	0,55 ± 0,27	0,56 ± 0,31	0,852
8	0,45 ± 0,26	0,48 ± 0,29	0,441
9	0,44 ± 0,24	0,43 ± 0,25	0,751
10	0,42 ± 0,23	0,44 ± 0,23	0,265
11	0,41 ± 0,24	0,42 ± 0,26	0,675
12	0,32 ± 0,14	0,38 ± 0,20	0,078

## Discussion

In the study, we analyzed fixation stability for each eye separately. When analyzing binocular fixation, the gaze position is calculated from the average gaze positions of the right and left eyes (12). Previous studies have indicated that binocular fixation becomes more stable with children age (3; 15), and therefore, we expected a similar trend in monocular fixation. Our results suggest that the fixation stability of each eye is related to the child's age. Fixation becomes more stable, especially up to the age of 9. This could be explained by the fact that this age is considered an approximate endpoint of visual system development (14). However, characteristics defining visual systems, such as contrast sensitivity, continue to develop significantly until the age of 12 (7), and hyperacuity even until the age of 21 (23).

The main aim of the study was to analyze whether there is a difference in fixation stability between the dominant and non-dominant eye in school-age children. Similar to other studies (16; 22) that compared fixation stability of the dominant and non-dominant eye in adults, no significant difference in fixation stability between the dominant and non-dominant eye was observed in children after the age of 9. However, the most significant finding of the study is that for 7 and 8-year-old children, fixation in the dominant and non-dominant eye differs significantly. When eye dominance is not considered, such differences in fixation stability are not observed when comparing the fixation stability of the right and left eye.

The dominant eye processes sensory information faster (18). It is more commonly observed that the dominant eye is the right one (5), and this was also evident among the participants in our study, as the dominant eye was most frequently the right one in all age groups. However, we cannot rule out unstable ocular dominance among study participants because the dominant eye was determined using one test and only once. In studies that analyze fixation stability by selecting the results of one eye, there is no uniform guideline for choosing which eye's data to use. As in the study of Vikesdal & Langaas (22) a difference in fixation stability between the dominant and non-dominant was observed, the authors suggest that in all studies requiring high-precision eye movement recordings, the dominant eye should be chosen. Based on the results of our study, we agree with this assertion, as both the dominant and non-dominant eyes may play a significant role in the analysis of fixation stability, especially in younger children, as the dominant eye shows more stable fixation than the non-dominant eye.

Studies indicate that minor refractive errors do not significantly affect fixation stability (21); however, the presence of amblyopia can impact fixation stability (1; 8). A minor limitation of our study is that the selection criteria did not completely exclude the possibility of amblyopic children participating in the study. However, research suggests that the prevalence of amblyopia in European countries is approximately 2.66% (10), which, overall, would constitute a small fraction of the total number of children participating in the study. Additionally, some children with amblyopia may not have met visual acuity criteria (24). The prevalence of amblyopia among children should be considered but it is not expected that mentioned limitation significantly affect our study's results.

## Conclusion

This study shows that for younger school-age children, significantly more stable fixation is observed in the dominant eye compared to the non-dominant eye. When fixation stability is analyzed using results from a one eye, for a more objective analysis, attention should be paid to eye dominance. Attention should also be paid to the age of the children, as monocular fixation becomes more stable as the children grow older.

In future studies, it would be valuable to determine how significantly fixation stability differs between the dominant and non-dominant eye in preschool-age children and whether unstable ocular dominance affects fixation stability.

### Ethics and Conflict of Interest

The author(s) declare(s) that the contents of the article are in agreement with the ethics described in http://biblio.unibe.ch/portale/elibrary/BOP/jemr/ethics.html and that there is no conflict of interest regarding the publication of this paper.

### Acknowledgements

We would like to express our gratitude to the schools participating in the study (Marupe State Gymnasium, Marupe Elementary School, Riga Cultures Secondary School, and Kuldiga Center Secondary School). The study is supported by the Latvian Council of Science (project No lzp-2021/1-0219, and SIA Mikrotikls and University of Latvia Foundation (project No 2260).
